# Cochleaimplantatmikrofoncheck mittels Datalogging

**DOI:** 10.1007/s00106-025-01652-x

**Published:** 2025-08-18

**Authors:** Tim Liebscher, Cynthia Glaubitz, Anne Hast, Ulrich Hoppe

**Affiliations:** Hals-Nasen-Ohrenklinik, Kopf- und Halschirurgie, Cochlear-Implant-Centrum CICERO, Uniklinikum Erlangen, FAU Erlangen-Nürnberg, Erlangen, Deutschland

**Keywords:** CI-Nachsorge, Qualitätssicherung, Sprachprozessor, Soundprozessor, Szenenerkennung, Cochlea implant aftercare, Quality control, Speech processor, Sound processor, Scene classification

## Abstract

**Hintergrund:**

In der Cochleaimplantat(CI)-Nachsorge ist die technische Überprüfung der internen und externen Komponenten ein fester Bestandteil. Routinemäßig wird der Sprachprozessor (SP) v. a. auf die volle Funktionsfähigkeit der Mikrofone hin geprüft. Abhängig vom SP können diese durch Fachpersonal abgehört oder in der CI-Anpasssoftware überprüft werden. Auch können Mikrofondefekte durch schlechtere Hörleistung aufgedeckt werden. Moderne CI-Systeme zeichnen via Datalogging auch die Dauer der Nutzung des SP und der Exposition bestimmter Hörumgebungen auf. Bei einer bilateralen CI-Versorgung könnten große Abweichung der erkannten Hörumgebung auf ein defektes Mikrofon hinweisen. Ziel dieser Arbeit ist es, die Datalog-Einträge von bilateralen CI-Trägern zu vergleichen, um potenzielle Mikrofondefekte zu identifizieren.

**Methodik:**

Die retrospektive Datenanalyse umfasst 359 bilaterale CI-Träger, die ein Cochlear-Nucleus-6-System oder höher erhalten haben. Die individuellen Datalog-Einträge wurden hinsichtlich der Dauer der CI-Nutzung sowie der Hörszenen „Ruhe“, „Sprache in Ruhe“, „Sprache im Störgeräusch“, „Lärm“ und „Musik“ ausgewertet und seitenweise verglichen. Bei einer vergleichbaren Nutzungsdauer werden Abweichungen von mehr als 15 % zwischen den Hörszenen des linken und des rechten Sprachprozessors als auffällig gewertet.

**Ergebnisse:**

Unter den 718 untersuchten Sprachprozessoren wurden 9 als auffällig bewertet. Bei gleicher Nutzungsdauer beider Sprachprozessoren konnten in der Hörumgebung „Ruhe“ signifikante Unterschiede von bis zu 54 % zwischen beiden Geräten nachgewiesen werden. Einer der Sprachprozessoren erkannte lediglich die Szene „Ruhe“ zu 100 %.

Anhand von 2 Fallbeispielen werden der Zeitraum der CI-Versorgung, das Trageverhalten der Patienten, die Hörumgebungen und die nachweisbaren Unterschiede zwischen den beiden Sprachprozessoren detailliert dargestellt.

**Schlussfolgerung:**

Die Informationen aus den Datalogs ermöglichen die Erkennung von möglichen Mikrofondefekten. Die Daten werden in der CI-Anpassung ohnehin automatisch ausgelesen und angezeigt. Es wird empfohlen, diese Informationen in die klinische Routine im Rahmen der technischen Überprüfung zu integrieren.

## Abstract (en)

**Background:**

The technical inspection of internal and external components is an integral part of cochlear implant (CI) aftercare. The speech processor (SP) is routinely checked to ensure that the microphones are fully functional. Depending on the SP, these can be listened to by specialist personnel or checked in the CI fitting software. Microphone defects can also be revealed by poor hearing performance. Modern CI systems also record the duration of use of the SP and exposure to certain listening environments via data logging. In a bilateral CI fitting, large deviations in the detected hearing environment between both processors could indicate faulty microphones. The aim of this study is to compare the data logging entries of bilateral CI users in order to identify potential microphone failures.

**Methodology:**

The retrospective data analysis includes 359 bilateral CI users who received a Cochlear Nucleus 6 system or higher. The individual data logging entries were analyzed with respect to the duration of CI use and duration of the listening scenarios quiet, speech in quiet, speech in noise, noise and music and both sides were compared. With a comparable duration of CI use, deviations of more than 15% between the listening scenarios of the left and right SP were considered conspicuous.

**Results:**

Of the 718 SPs examined 9 were rated as conspicuous. With a comparable CI use of the left and right SP, significant differences of up to 54% were detected between the two devices in the quiet listening environment. One of the SPs recognized only the quiet scene to 100%. Based on two case examples the period of the patient’s wearing behavior, the listening environments and the detectable differences between the two individual SPs during CI aftercare are presented in detail.

**Conclusion:**

The information from the data logging enables the detection of possible microphone defects. The data are automatically read out and displayed anyway during the CI fitting. It is recommended to integrate the data logging information into the clinical routine as part of the technical check.

## Hintergrund und Fragestellung

Seit mehr als 10 Jahren bieten Cochleaimplantat(CI)-Systeme die Möglichkeit, das Nutzerverhalten des Patienten aufzuzeichnen. Mit Hilfe der Datalogging-Funktion können z. B. Daten wie die Dauer der CI-, der Programm- und der Zubehörnutzung sowie Informationen über die akustische Hörumgebung wie etwa der mittlere Schallpegel über einen bestimmten Zeitraum gespeichert und bei der CI-Anpassung als Übersicht dargestellt werden. Systembedingt werden je nach CI-Hersteller und Prozessorgeneration verschiedene Datalog-Einträge erhoben (Tab. 1). In der klinischen Routine werden diese Daten zur individuellen Beratung genutzt. Auffälliges Nutzerverhalten wie z. B. geringe Nutzungsdauer, ungeeignete Programmwahl, Lautstärkeeinstellung oder Hörumgebung kann so erkannt und in der CI-Therapie gezielt angesprochen werden.

**Tab. 1:** Auszug der Datalog-Einträge der Cochleaimplantat(CI)-Hersteller Advanced Bionics, Cochlear und MED-EL aus ihren aktuellsten (Stand 02/2025) Sprachprozessoren (Naída/Sky CI M, N8/Kanso2 bzw. Sonnet 3/Rondo 3); die Verfügbarkeit der Daten kann je nach Sprachprozessormodel und CI-Einstellungen variieren

	Advanced Bionics	Cochlear	MED-EL
Log-Periode (in Tagen)	x	x	x
Prozessornutzungsdauer h/Tag	x	x	x
Einschaltvorgänge/Tag	–	x	x
Kontaktunterbrechung	x	x	x
Programmnutzungsdauer h/Tag	x	x	x
Szenen bzw. automische Programme	Ruhige Umgebung Verstehen im Störgeräusch Verstehen bei Fahrgeräusch Komfort im Störgeräusch Komfort in halligen Situationen Musik	Sprache Sprache im Störgeräusch Ruhe Musik Störgeräusch	Sprache Sprache im Störgeräusch Ruhe Musik Störgeräusch
Umgebungsschallpegel	–	<40 dB(A) 40–50 dB(A) 50–60 dB(A) 60–70 dB(A) 70–80 dB(A)	≤45 dB (SPL) 46–55 dB (SPL) 56–65 dB (SPL) 66–75 dB (SPL) 76–85 dB (SPL) ≥86 dB (SPL)
Änderungen von Lautstärke/Mikrofonempfindlichkeit	–	x	x
AUX/Zubehör	Streaming (Smartphone) T‑Coil RogerDirect Partner Mic	Streaming (Smartphone) T‑Coil Roger 20 MiniMic (2+) Phone Clip TV Streamer	Streaming (Smartphone) T‑Coil Roger 21/AUX AudioLink/XT

*SPL* „sound pressure level“, *AUX* „auxiliary“

Die Bedeutung einer ausreichend hohen täglichen CI-Nutzungs-Dauer sowie einer adäquaten Hörumgebung für die Hör- und Sprachentwicklung bei Kindern konnte bereits in verschiedenen Studien nachgewiesen werden [1–3]. Auch bei erwachsenen CI-Trägern konnte gezeigt werden, dass neben dem präoperativen Sprachverstehen die tägliche Nutzungssauer des CI ein signifikanter Faktor für das Sprachverstehen mit CI ist [4]. Lindquist et al. (2023) empfehlen eine Nutzungsdauer von mindestens 12 h pro Tag. Entsprechend wichtig ist die Auswertung der individuellen Datalog-Einträge.

In multizentrischen Studien wurden neben der Nutzungsdauer auch die Hörumgebungen von mehreren tausend CI-Trägern unterschiedlichen Alters untersucht [5–7]. Diese Daten verdeutlichen die unterschiedlichen Hörumgebungen der jeweiligen Altersgruppen. Im Gruppenvergleich sind beispielsweise Kleinkinder am häufigsten der Hörumgebung „Musik“, Schulkinder der Hörumgebung „Sprache“, junge Erwachsene der Hörumgebung „Lärm“ und Senioren der Hörumgebung „Ruhe“ ausgesetzt. Die Situation „Sprache im Störgeräusch“ wurde im Gruppenvergleich für Schulkinder am häufigsten bestimmt.

Diese Hörumgebungen werden durch eine Szenenanalyse klassifiziert. Bei den CI-Systemen des Herstellers Cochlear (Cochlear Ltd.) ist diese Teil des SCAN(„scene analysis“)-Algorithmus [8], der seit der Markteinführung des Nucleus-6-Systems im Jahr 2013 fest implementiert ist. Dieser Algorithmus analysiert das Mikrofoneingangssignal hinsichtlich spezifischer Signalmerkmale wie der Modulation mit Musik, Sprache- oder Störgeräuschen und klassifiziert diese entsprechend den 5 möglichen Hörumgebungen „Sprache in Ruhe“, „Sprache im Störgeräusch“, „Lärm“, „Ruhe“ und „Musik“. Je nach Einstellung des CI-Programms werden dann Mikrofoncharakteristik, Mikrofonempfindlichkeit und Störgeräuschunterdrückung automatisch eingestellt, um daraus die beste Signalvorverarbeitung zu einzustellen.

Mit Blick auf die mittleren Ergebnisse der multizentrischen Studien deutet eine ungewöhnlich hohe oder auch niedrige Exposition bestimmter Hörumgebungen entweder auf eine ungewöhnliche individuelle Höranforderung bzw. auf ein ungewöhnliches individuelles Trageverhalten hin, es könnte aber auch ein Hinweis auf einen Defekt im Mikrofon des Sprachprozessors sein. Werden z. B. über einen bestimmten Zeitraum die Szene „Ruhe“ zu 100 % und dementsprechend alle anderen Szenen gar nicht erkannt, liegt mutmaßlich ein technischer Defekt vor. Ein defektes Sprachprozessormikrofon zeichnet dann ein unzureichendes Eingangssignal auf, welches zu einer Fehlklassifizierung der Szene führt.

Voraussetzung für eine solche Bewertung ist ein ausreichend langer Datalog-Eintrag mit einer hohen durchschnittlichen Nutzungsdauer, um das durchschnittliche tägliche Trageverhalten und die individuelle Hörumgebung widerzuspiegeln. Bei beidseitig versorgten CI-Trägern kann auch der Vergleich beider Seiten herangezogen werden. Liegt bei einer vergleichbaren Nutzungsdauer beider Sprachprozessoren eine große Abweichung der Hörumgebung links und rechts vor, könnte diese Differenz auf einen Sprachprozessordefekt hinweisen.

Die technische Integrität des implantierten Systems einschließlich aller externen Komponenten ist integraler Bestandteil der CI-Versorgung. Gemäß den Empfehlungen der Deutschen Gesellschaft für Audiologie sind technische Überprüfungen regelmäßig durchzuführen und umfassen die Funktionsprüfung des Sprachprozessors inklusive des Abhörens der Mikrofone und einer objektiven Kontrolle (z. B. Messbox). Darüber hinaus sieht diese Empfehlung eine objektive Prüfung der Sprachprozessormikrofone vor und bewertet auch diesen Arbeitsschritt als notwendig [9]. Eine objektive Überprüfung der spezifikationsgerechten Funktion aller Mikrofone und externen Eingänge kann jedoch bei den meisten CI-Systemen nicht durchgeführt werden, da von den CI-Herstellern keine geeigneten Testsysteme zur Verfügung gestellt werden.

Ziel dieser Arbeit ist es, die Datalog-Einträge von bilateral versorgten CI-Trägern der Firma Cochlear individuell zu vergleichen und signifikante Abweichungen der aufgezeichneten Hörumgebung zu ermitteln, um mögliche Mikrofondefekte aufzudecken.

## Methodik

Die vorliegende Datenanalyse erfolgte retrospektiv. Ein positives Ethikvotum (Nr. 199_17 Bc) der Ethik-Kommission der Friedrich-Alexander-Universität Erlangen-Nürnberg liegt vor.

### Datenerhebung

Diese Datenanalyse betrachtet alle CI-Träger, die sich in der Nachsorge im CI-Centrum CICERO in Erlangen befanden und die mit einem Nucleus-6-System oder höher versorgt waren (Stand 06/2024). Die Datalog-Einträge jedes CI-Patienten und jeder CI-Seite wurden über eine eigens dafür programmierte SQL(„structured query language“)-Abfrage via MATLAB (2019b; The Mathworks Inc., Natick, MA, USA) abgerufen. Ein Datalog-Eintrag protokollierte Daten, welche innerhalb von 2 aufeinanderfolgenden Anpassungssitzungen erhoben wurden. Neben den Metadaten wie Zeitstempel (Datalog-Start- und -Endzeitpunkt), CI-Seite, Sprachprozessortyp beinhalten diese die CI-Nutzungs-Dauer.

**Abb. 1:**
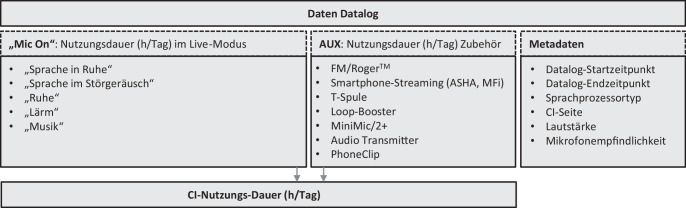
Auszug der exportierten Daten aus einem Datalog-Eintrag eines Sprachprozessors: Die durchschnittliche tägliche Cochleaimplantat(*CI*)-Nutzungs-Dauer setzt sich aus der Nutzungsdauer der Sprachprozessormikrofone (Live-Modus) sowie der Nutzungsdauer der *AUX*(„auxiliary“)-Schnittstellen zusammen. Im Live-Modus wird zusätzlich die Dauer der Hörszenen „Sprache in Ruhe“, „Sprache im Störgeräusch“, „Ruhe“, „Lärm“ und „Musik“ getrennt aufgezeichnet. Ein Datalog-Eintrag beschreibt den Zeitraum zwischen 2 Anpasssitzungen (*ASHA* „audio streaming for hearing aids“, *MFi* „made für iPhone“, *FM* frequenzmodulierte Funksignale)

Die Nutzungsdauer der Sprachprozessormikrofone ergibt sich aus der Summe der Exposition der automatisch klassifizierten 5 Hörsituationen „Sprache in Ruhe“, „Sprache im Störgeräusch“, „Ruhe“, „Lärm“ und „Musik“ in Stunden pro Tag (h/Tag). Außerdem wird die Nutzungsdauer des Zubehörs aufgezeichnet. Die Gesamtdauer der CI-Nutzung setzt sich aus der Dauer der Nutzung der Sprachprozessormikrofone und des Zubehörs zusammen. Abb. 1 beschreibt den Zusammenhang.

### Patientenkollektiv

**Abb. 2:**
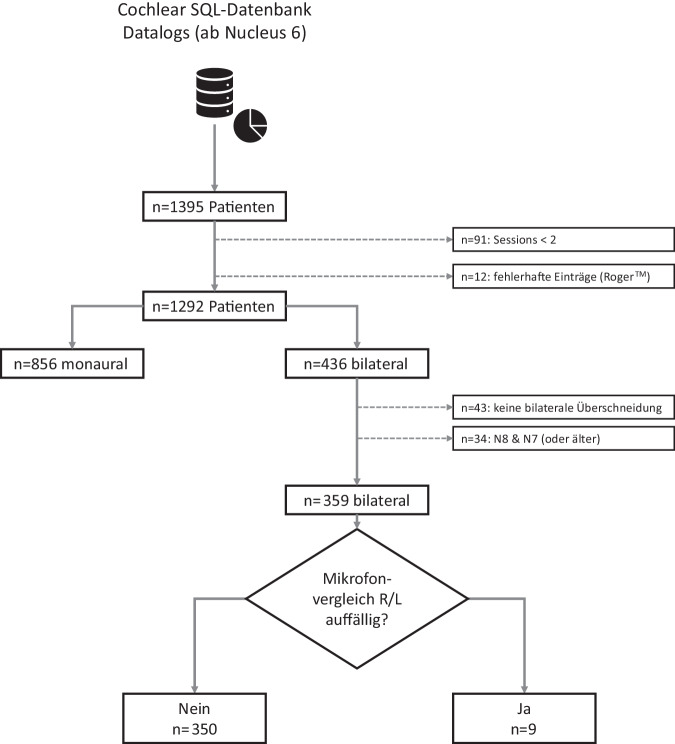
Flussdiagramm der Studie: Verteilung der bilateralen Cochleaimplantatträger mit verwertbaren Datalog-Einträgen (*SQL* „structured query language“, *R* rechts, *L* links)

Insgesamt lagen 163.217 Log-Einträge von 1395 CI-Trägern mit 1871 CI vor. Im Folgenden wurden CI-Seiten ausgeschlossen, bei denen insgesamt weniger als 2 Datalog-Einträge (Sessions) vorhanden sind, da wegen fehlender Daten kein bilateraler Vergleich möglich ist. Exkludiert wurden zudem Patienten, die Roger^TM^-20-Empfänger (Sonova AG, Stäfa, Schweiz) in Kombination mit N8-Sprachprozessoren nutzen, da es zu irregulären Log-Einträgen in der Custom Sound Pro 7.0 (Build 7.0.090.118) kommen kann. Von den verbliebenen 1292 Patienten sind 856 monaural und 436 bilateral versorgt (Abb. 2).

Um die aufgezeichneten Szenen des rechten und linken Sprachprozessors des bilateralen Kollektivs zu vergleichen, ist es zudem notwendig, dass die Zeitstempel der Log-Einträge beider CI-Seiten vergleichbar sind. Dafür wurden die Start- und Endzeitpunkte der Log-Einträge beider Sprachprozessoren abgeglichen. Bei 43 Patienten gab es keine bilaterale Überschneidung der aufgezeichneten Zeiträume, weshalb für diese Gruppe kein bilateraler Szenenvergleich durchgeführt werden konnte.

Nicht weiter ausgewertet wurden zudem 34 Patienten, die zuletzt CI-Sprachprozessoren unterschiedlichen Typs (N8- und gegenseitig CP1000 oder älter) verwendet haben, da die Szenenerkennung (Scan bzw. Scan2) auf unterschiedlichen Algorithmen beruht.

Insgesamt wurden 359 bilateral versorgte Patienten in die Auswertung einbezogen. Die Patientenselektion ist in Abb. 2 zusammengefasst. Tab. 2 enthält die demografischen Daten der der eingeschlossenen Patienten. Die Verteilung der verwendeten Sprachprozessortypen ist in Tab. 3 dargestellt.

**Tab. 2:** Demografische Daten der 359 bilateralen Cochleaimplantat(*CI*)-Träger

	Median	Mittelwert	Min	Max
Alter bei 1. Operation (Jahre)	4,17	22,62	0,39	82,87
Alter bei 2. Operation (Jahre)	5,77	24,40	0,50	85,21
Abstand der beiden Versorgungen (Jahre)	0,74	1,77	0,00	17,45
Alter Datalog 1 (Jahre)	9,74	25,54	0,51	85,34
Alter Datalog 2 (Jahre)	18,28	31,55	1,24	91,67
CI-Erfahrung Seite 1 (Jahre)	6,89	6,15	0,27	19,65
CI-Erfahrung Seite 2 (Jahre)	8,74	8,24	0,38	24,62

**Tab. 3:** Verteilung der getesteten Sprachprozessoren und Sprachprozessoralter zum Zeitpunkt der letzten Testung

Sprachprozessor	CP910/920	CP50 (Kanso 1)	CP1000	CP1150 (Kanso 2)	N8 (CP1110)
Anzahl	126	43	394	25	130
*Sprachprozessoralter*					
Mittelwert (Jahre)	3,5 ± 2,8	4,2 ± 2,0	2,5 ± 1,6	1,1 ± 0,7	0,7 ± 0,5
Maximum (Jahre)	10,1	7,7	6,5	2,5	1,7
Minimum (Jahre)	0,1	0,4	0,1	0,1	0,1

Bei jeder CI-Kontrolle wurde das CI-System stets auf Probleme hinsichtlich der internen oder externen Hardware getestet. Hierzu wurden die Mikrofone des Sprachprozessors durch geschultes Personal abgehört. Es wurde zudem stets erfragt, ob die Mikrofonfilter in dem vom CI-Hersteller geforderten Zeitraum ausgetauscht wurden. Falls nicht, wurden diese vor Ort gewechselt.

### Bilateraler Vergleich

Um die Szenen zweier Sprachprozessoren zu vergleichen, muss sichergestellt werden, dass beide Sprachprozessoren auch der gleichen Hörumgebung ausgesetzt sind. Als Kenngröße wird hierfür die Nutzungsdauer beider Sprachprozessormikrofone herangezogen. Ist die Nutzungsdauerdifferenz zwischen rechtem und linkem Sprachprozessor kleiner als ein kritischer Wert *K*_*Nutzung*_ von 10 %, gehen wir von einem vergleichbaren Trageverhalten beider Sprachprozessoren aus. Dadurch wird ausgeschlossen, dass ein Sprachprozessor deutlich geringer verwendet wurde, was *per se* schon zu unterschiedlichen Hörszenen geführt hätte.

Ist die Nutzungsdauer vergleichbar, wird anschließend ein Szenenvergleich durchgeführt. Zum Vergleich der Szenen („Ruhe“, „Sprache in Ruhe“, „Sprache im Störgeräusch“, „Lärm“, „Musik“) werden die relativen Expositionen im Bezug zur Mikrofonnutzungsdauer berechnet. Die Differenz der Szenenrelativwerte zwischen rechtem und linken Sprachprozessor wird berechnet und abschließend bewertet. Liegt die Abweichung *K*_*Szene*_ zwischen beiden Geräten in mindestens 1 Szene über 15 %, wird der Vergleich als auffällig gewertet. Durch die Festlegung dieses Schwellenwerts wird sichergestellt, dass etwaige kurzzeitige Unterschiede der Akustik zwischen linkem und rechtem Sprachprozessor keinen Einfluss auf die Erkennung auffälliger Sprachprozessoren hat.

## Ergebnisse

Die Auswertung der 359 bilateralen CI-Träger zeigte 9 Fälle, bei denen signifikante Unterschiede in der Szenenanalyse aufgetreten sind. Unter den 718 Sprachprozessoren im Umlauf waren entsprechend 9 „auffällig“, das entspricht 1,25 %; 2 CP910, 1 CP950 und 6 CP1000 wurden identifiziert. Die „Ausfallrate“ je Sprachprozessormodell liegt in diesem Testkollektiv damit bei 1,6 % (CP910), 2,3 % (CP950) und 1,5 % (CP1000). Keiner der CP1150- oder CP1110-Prozessoren war auffällig.

### Fallbeispiel 1

**Abb. 3:**
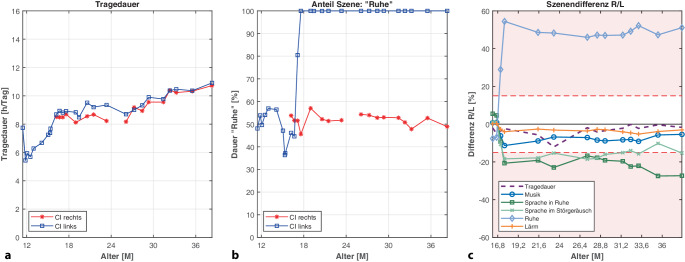
Fallbeispiel 1 – Kind: Erstversorgung links im Alter von 10,5 Monaten, nach 4 Monaten Cochleaimplantat(*CI*)-Versorgung rechts (*R* rechts, *L* links, *M* Monate): **a** tägliche Nutzungsdauer beider im Verlauf altersentsprechend und im Verlauf beidseitig steigend, im Alter von 3 Jahren durchschnittliche Nutzungsdauer beidseits bei 10 h/Tag; **b** Sprachprozessor links erkennt nach ca. 6 Monaten die Szene „Ruhe“ zu 100 %; **c** signifikante Unterschiede (*K*_*Szene*_ > 15 %, *hellroter Bereich*) für die Szenen „Sprache in Ruhe“, „Sprache im Störgeräusch“ und „Ruhe“

Abb. 3 zeigt den Verlauf der Datalog-Daten eines sequenziell bilateral versorgten Kindes. Die Erstversorgung rechts erfolgte im Alter von 10,5 Monaten, die Implantation links 4 Monate später. Das Kind war beidseits mit CP1000-Sprachprozessoren versorgt. Die tägliche CI-Nutzungs-Dauer war beidseits vergleichbar hoch (Abb. 3a). Im Patientenalter von etwa 17 Monaten betrug sie durchschnittlich 8 h/Tag. Bis zum Ende des dritten Lebensjahrs stieg die CI-Nutzungs-Dauer beidseitig auf rund 10 h/Tag an.

Der Anteil der Szene „Ruhe“ (Abb. 3b) wurde mit dem rechten Sprachprozessor mit durchschnittlich 51 % erkannt. Der linke Sprachprozessor erkannte „Ruhe“ in den ersten 6 Monaten mit durchschnittlich 49 %. In dem darauffolgenden Zeitraum (> 17 Monate) wurde diese Szene links dann konstant mit 100 % erkannt. Dementsprechend wurden die restlichen Szenen links („Sprache in Ruhe“, „Sprache im Störgeräusch“, „Lärm“ und „Musik“) zu 0 % erkannt.

Die relative Differenz der Szenen und der Nutzungsdauer zwischen linkem und rechtem Sprachprozessor ist in Abb. 3c dargestellt. Die Nutzungsdauer zwischen den beiden CI-Seiten unterschied sich im Mittel um 3,3 % (SD [„standard deviation“] = ±2,9 %; Range = 0,1–12,1 %). Zum frühestmöglichen Vergleichszeitpunkt im Alter von 17 Monaten betrug die Differenz der Szenen „Sprache in Ruhe“, „Sprache im Störgeräusch“, „Lärm“, „Ruhe“ und „Musik“ zwischen beiden Sprachprozessoren maximal 7,6 %. In den folgenden Zeiträumen traten deutliche Unterschiede bis zu 54 % („Ruhe“), 27 % („Sprache in Ruhe“), 18 % („Sprache im Störgeräusch“), 11 % („Musik“) und 5,3 % („Lärm“) auf.

Im Rahmen der routinemäßigen Techniküberprüfungen während der mehrjährigen Folgetherapie wurden keine Anzeichen für einen technischen Defekt festgestellt. Die Funktion der Sprachprozessoren und der Mikrofone wurde bei jeder Anpasseinheit überprüft. Das Abhören der Mikrofone mit dem Nucleus-7-Kontrollkopfhörer (Cochlear Monitor Earphone Adaptor) war stets unauffällig. Die Freifeldtonaudiometrie zeigte seitengetrennt altersgerechte Aufblähkurven. Die Vorverarbeitung „SCAN“ war zudem stets deaktiviert. Nach dieser Analyse wurde der Austausch des linken Sprachprozessors eingeleitet. Nach der ersten Wiedervorstellung zeigten sich wieder eine vergleichbare Szenenanalyse und vergleichbare Hörumgebungen auf beiden Seiten.

### Fallbeispiel 2

**Abb. 4:**
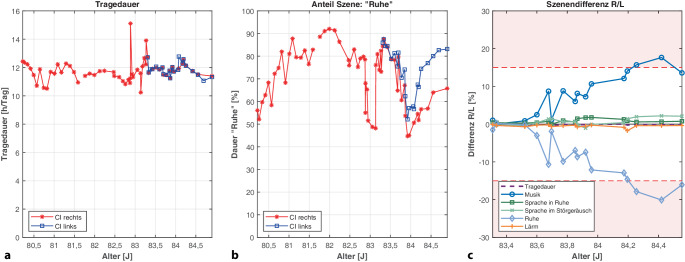
Fallbeispiel 2 – Erwachsener Cochleaimplantat(*CI*)-Träger (Erstversorgung rechts im Alter von 80,2 Jahren, nach 3 Jahren CI-Versorgung links *(R* rechts, *L* links, *J* Jahre): **a** Tägliche Nutzungsdauer beider CI durchschnittlich bei 11,8 h/Tag. **b** 6 Monate nach der bilateralen Versorgung wird die Szene „Ruhe“ links zunehmend häufiger als rechts erkannt. **c** Die Differenz der einzelnen Szenen zeigt keine signifikanten Unterschiede für die Szenen „Sprache in Ruhe“, „Sprache im Störgeräusch“ und „Lärm“; bei „Musik“ und „Ruhe“ liegen die Differenzen jeweils über dem kritischen Wert (*K*_*Szene*_) von 15 % (*hellroter Bereich*)

Das CI-Trageverhalten eines erwachsenen CI-Trägers ist in Abb. 4 dargestellt. Der Patient wurde im Alter von 80,2 Jahren links und von 83,2 Jahren rechts mit CI versorgt. Der Patient trug beidseits CP1000-Sprachprozessoren.

Die CI-Nutzungs-Dauer betrug links und rechts im Durchschnitt 11,8 h/Tag (Abb. 4a). Der Anteil der Szene „Ruhe“ (Abb. 4b) betrug links durchschnittlich 70,6 % (SD = ±13,3 %; Range = 44,7–92,0 %) und rechts 73,7 % (SD = ±10,8 %; Range = 52,1–87,4 %). In den letzten Monaten wurde die Szene „Ruhe“ dann links häufiger (zuletzt 83,2 %) erkannt als rechts (65,8 %).

Der direkte Vergleich der Nutzungsdauer (Abb. 4c) ergab Differenzen von nur 0,1 % (SD = ±0,1 %; Range = 0–0,6 %). Beide Sprachprozessoren wurden gleichermaßen häufig getragen. Die Differenz der Szenen „Sprache in Ruhe“, „Sprache im Störgeräusch“ und „Lärm“ zwischen beiden Sprachprozessoren lagen bei maximal 1,8, 3,2 bzw. 1,7 %. Hingegen wurden links die Szene „Ruhe“ um 17 % häufiger und die Szene „Musik“ um 20 % seltener erkannt als mit dem rechten Sprachprozessor.

Auch im Fallbeispiel 2 gab es während der CI-Nachsorge keine Anzeichen auf einen Defekt. Der Patient klagte aber stets über schwankende Hörleistungen. Auf beiden Sprachprozessoren war der SCAN-Algorithmus aktiviert. Durch ungleiche Verteilung der Szenen „Musik“ und „Ruhe“ in Kombination mit dem SCAN-Algorithmus war nicht sichergestellt, dass die Mikrofoncharakteristik und die Verstärkung des rechten und linken Sprachprozessors gleichermaßen eingestellt werden. Daher wurden beide Sprachprozessoren ausgetauscht.

## Diskussion

Die Untersuchungsergebnisse der Cochlear-Datalog-Einträge des großen bilateral versorgten Patientenkollektivs zeigen, dass es in einigen Fällen zu großen Abweichungen der automatischen Szenenerkennung zwischen beiden Sprachprozessoren kommt. Die Ursache für diese Unterschiede kann durch technische Defekte der Sprachprozessoren erklärt werden, die evtl. einen Austausch erfordern.

Mikrofondefekte können unterschiedlich stark aus- und auffallen. Im Extremfall nehmen Mikrofone keinen verwertbaren Nutzschall mehr auf oder geben ein Rauschen oder Brummen wieder. Diese Defekte lassen sich i. d. R. durch Abhören der Mikrofone, durch Rückmeldung des Patienten oder im Hörtest erkennen. Je nach Sprachprozessormodell oder Patienten-Compliance ist diese Überprüfung aber oft gar nicht möglich.

Ein weniger deutlicher Mikrofondefekt kann von Patienten sowie Audiologen ggf. gar nicht identifiziert werden. Gibt es keine Rückmeldung vom Patienten und sind die ton- und sprachaudiometrischen Freifeldmessungen in Ruhe stabil, gibt es augenscheinlich keinen weiteren Handlungsbedarf. Die vorliegenden Ergebnisse zeigen jedoch, dass bei mutmaßlichen Mikrofondefekten abweichende Hörumgebungen zwischen beiden Sprachprozessoren mit der Szenenanalyse erkannt werden. Nutzt der Patient das SCAN-Programm beidseitig, hat dies zur Folge, dass Mikrofoncharakteristik, Mikrofonempfindlichkeit sowie Störgeräuschunterdrückung links und rechts zu unterschiedlichen Zeitpunkten und/oder unterschiedlich lang eingestellt werden. Inwieweit diese bilaterale Differenz auch eine binaurale Interferenz bedingt, ist unklar. Im Zweifel sollten beide Sprachprozessoren vom CI-Hersteller überprüft werden.

### Besonderheiten der Methodik und Limitationen

Ein Datalog-Eintrag wird über einen bestimmten Zeitraum, meist mehrere Wochen oder Monate, aufgezeichnet. Der hier vorgestellte Ansatz setzt voraus, dass beide Sprachprozessoren zu gleichen Zeitpunkten getragen werden, damit die Szenenanalyse auch dieselbe Hörumgebung aufzeichnet. Die Nutzungsdauer beider Sprachprozessoren ist aktuell der einzige Indikator zur Einordnung des bilateralen Nutzungsverhaltens. Für den unwahrscheinlichen Fall, dass ein Patient beispielsweise tage- oder wochenweise wechselseitig nur einen Sprachprozessor trägt, würden zwar eine vergleichbare Nutzungsdauer, aber wohl unterschiedliche Hörumgebungen aufgezeichnet werden.

Benutzt der CI-Träger die Nucleus Smart App regelmäßig und ist Cochlear Remote Check verfügbar, wird die Nutzungsdauer auch detaillierter angezeigt [10]. Dann liegen die Log-Daten der täglichen Nutzung vor, und der Abgleich beider Sprachprozessoren wäre wesentlich genauer. Diese Daten sind aber aktuell nur für registrierte Patienten und nur auf Anfrage der Klinik beim Patienten erhältlich.

Treten Unstimmigkeiten bei der Betrachtung der Datalog-Einträge auf, sollte auch stets geprüft werden, ob zuletzt ein Sprachprozessortausch (Defekt oder Ausprobe), ein Besuch in einer anderen Klinik oder bei einem anderen CI-Servicepartner stattgefunden hat. Werden nicht alle Sprachprozessoren bei den Anpassungen konsistent angeschlossen, die Datalogs ausgelesen und zurückgesetzt sowie die CI-Anpassdaten abgeglichen, gibt es womöglich Leereinträge, oder die CI-Nutzung wird einem falschen Zeitraum zugeordnet und damit falsch dargestellt.

Obwohl die hier präsentierten Daten ausschließlich mit CI-Systemen des Herstellers Cochlear (Cochlear Ltd.) erhoben und ausgewertet wurden, ist davon auszugehen, dass ähnliche Effekte auch bei anderen CI-Herstellern und Hörsystemen auftreten können. Je nach Sprachprozessor ist es ggf. nötig, andere Parameter wie „Dauer der automatischen Programme“ oder „Umgebungsschallpegel“ in die individuelle Auswertung einzubeziehen (siehe Tab. 1).

Mit den hier dargelegten, retrospektiv erhobenen Daten kann nicht eindeutig geklärt werden, was zu diesen Szenenabweichungen geführt hat. Der Großteil der Patienten hatte auch mit diesem Sprachprozessordefekt, soweit beurteilbar, adäquate bzw. stabile Hörleistungen. Möglich ist, dass nur eines der beiden Sprachprozessormikrofone außerhalb der Spezifikationen arbeitet, was dann zu einer Abweichung der detektierten Hörszenen führt.

Diese und andere Mikrofonabweichungen können auch bei Programmen ohne SCAN-Automatik, aber mit voreingestellter Mikrofoncharakteristik, zu Funktionseinschränkungen führen. Der Grund ist, dass dem Beamformer zur Einstellung der Mikrofonrichtcharakteristik dann möglicherweise falsche Einstellparameter zur Verfügung gestellt werden. Ob auch schleichende Mikrofonabweichungen mittels Datalog erfasst werden können, ist aktuell unklar. Für eine umfassende objektive Bewertung der CI-Sprachprozessormikrofone wäre der Einsatz einer Messbox, wie sie in der Hörgeräteanpassung Verwendung findet, notwendig und aus klinischer Sicht wünschenswert.

### Anwendung bei monauraler CI-Versorgung

Auch wenn die hier vorgestellt Studie bilaterale CI-Träger beleuchtet hat, kann eine genauere Betrachtung der individuellen Datalogs auch bei monauraler CI-Versorgung helfen, Mikrofondefekte ausfindig zu machen. Weicht die Hörszenenerkennung sehr stark von früheren Datalogs des Patienten oder von denen des Normkollektivs [5–7] ab, könnte auch das ein Hinweis auf ein defektes Sprachprozessormikrofon sein. Das Fallbeispiel 1 (Abb. 3b) zeigt deutlich, dass bereits bei der einzelnen Betrachtung des linken Sprachprozessors durch die Registrierung der Szene „Ruhe“ zu 100 % der Sprachprozessor nicht mehr innerhalb der Spezifikationen arbeitet. Solche sprunghaften Veränderungen der Szenenklassifikation sollten daher zu einer genaueren technischen Kontrolle führen.

## Schlussfolgerung und Ausblick

Datalogs können neben dem Nutzungsverhalten auch technische Fehler aufzeigen, wenn bestimmte Hörumgebungen ungewöhnlich häufig registriert werden. Der direkte Vergleich beider Sprachprozessoren bei bilateraler Versorgung hilft dabei, Abweichungen bei der Hörszenenerkennung zu identifizieren. Im Zweifel sollte der CI-Hersteller zur Prüfung der Sprachprozessoren herangezogen werden.

Neuerdings bieten CI-Hersteller in speziellen Apps auch die Möglichkeit, einen Systemcheck durchzuführen, bei dem die integrierten Sprachprozessormikrofone untereinander abgeglichen werden. Treten signifikante Abweichungen auf, bekommen der Audiologe und/oder der Patient einen entsprechenden Hinweis. Wie genau diese Überprüfungen ablaufen, wie häufig und v. a. wie valide diese sind, ist zum aktuellen Zeitpunkt noch nicht geklärt. Sind beispielsweise beide Mikrofone eines Sprachprozessors verschmutzt, wird womöglich keine Signaldifferenz angezeigt. Die Datalog-Analyse könnte hier Abhilfe schaffen.

## Fazit für die Praxis

- Cochleaimplantat(CI)-Hersteller bieten unterschiedliche Möglichkeiten an, die Sprachprozessormikrofone zu überprüfen. Eine allumfassende objektive Kontrollmöglichkeit für alle Sprachprozessormodelle gibt es aktuell nicht.
- Die im Datalog aufgezeichneten Hörumgebungen können helfen, technische Fehler des CI-Sprachprozessors zu erkennen, wenn einzelne Hörumgebungen vergleichsweise ungewöhnlich häufig registriert werden.
- Bei bilateraler CI-Versorgung mit vergleichbarer Nutzungsdauer beider Sprachprozessoren können große Unterschiede in den aufgezeichneten Szenen zwischen linkem und rechtem Sprachprozessor auf Mikrofondefekte hinweisen.
- Auch bei monauraler CI-Versorgung sind dominante Szenenanteile von 100 % als kritisch zu werten.

## Data Availability

Die in dieser Studie erhobenen Datensätze können auf begründete Anfrage beim Korrespondenzautor angefordert werden.
